# Neuropsychological assessment in MS is outdated and is in need for innovation: Yes

**DOI:** 10.1177/13524585241230184

**Published:** 2024-01-30

**Authors:** Pauline T Waskowiak, Marit FL Ruitenberg, Hanneke E Hulst

**Affiliations:** MS Center Amsterdam, Department of Medical Psychology, Vrije Universiteit Amsterdam, Amsterdam Neuroscience, Amsterdam UMC Location VUmc, Amsterdam, The Netherlands; Health, Medical and Neuropsychology Unit, Leiden University, Leiden, The Netherlands; Leiden Institute for Brain and Cognition, Leiden University, Leiden, The Netherlands; Health, Medical and Neuropsychology Unit, Leiden University, Leiden, The Netherlands; Leiden Institute for Brain and Cognition, Leiden University, Leiden, The Netherlands

Up to 65% of people with multiple sclerosis (PwMS) experience cognitive symptoms such as slower information processing and memory deficits.^1^ These can develop already early in the disease, and negatively impact daily functioning, work ability, and overall quality of life.^1–3^ To evaluate cognitive performance and detect cognitive dysfunction, neuropsychological assessment (NPA) is used. This typically consists of paper-and-pencil tests that find their roots several decades ago.^4^ That brings us to our primary question: is the currently used NPA old-fashioned and in need for innovation?

There are several challenges with NPAs that are currently used for PwMS (e.g. Brief International Cognitive Assessment for MS;^5^ Minimal Assessment of Cognitive Function in Multiple Sclerosis^6^). First, they require the involvement of specialized personnel (and subsequently money due to higher health care costs), hindering frequent and structural assessment of cognitive functioning and thus early identification of cognitive decline.^2^ Second, the long duration of these assessments can be particularly burdensome for PwMS who experience fatigue and/or mood problems, such that test scores for this group may not reflect true cognitive abilities. A third, more practical limitation of current NPAs in MS is that they are often unsuitable for annual retest assessments, due to learning effects and limited number of parallel versions. This is especially problematic for the California Verbal Learning Test–second edition, frequently used to assess verbal memory in PwMS, as learning effects are generally strong for this domain and only two parallel versions exist.^7^ Given the observed increase in overall performance on memory tests over time (in line with the *Flynn-effect*, i.e. increase in IQ scores), the interpretation of follow-up test scores may be unreliable.^8^ This may lead to an overestimation of cognitive functioning, which would be especially concerning for PwMS with subtle cognitive impairments. Fourth, norm scores are often outdated originating from a period in which Internet was unavailable and society functioned in a different way. As such, norms are in dire need for updating. Finally, NPA outcomes only marginally inform us about daily functioning and the lack of ecological validity is a sincere concern.^4^

Many of the challenges described above can be overcome by digitalizing NPA. For instance, the administration of a test battery and automated scoring without involvement of a test leader would not only facilitate regular cognitive screening in MS, but it would also avoid human error in its scoring.^9^ However, as digitalizing NPA is basically taking the old-fashioned test and putting on a new jacket, one can argue that within the field of neuropsychology we need to (and can) do better than that.

There has been relatively little progress in the field of neuropsychology (especially when compared to the rapid advances in other fields of MS, such as ongoing developments in neuroimaging technology to monitor disease activity). This may be a result of the hard work and dedication put into developing, validating, and standardizing tests, having professionals relying on their well-known instruments. However, technological advancements can bring NPA for PwMS (and beyond) to the next level once new test paradigms and scoring methods are developed that align with cognitive requirements of today’s society. For instance, computerized assessments allowing for computer adaptive testing (CAT), where the test adapts to the ability level of the patient, will enable more sophisticated assessment, for example, fitting with a person’s work requirements.^4^ As a nice side-effect, assessments can be more efficient and less burdensome for both PwMS and health care providers. Furthermore, technological solutions that allow for different measurements compared to what we have available today, like easy adjustment of stimuli and test versions, higher standardization, and inclusion of additional metrics (e.g. pencil pressure, the number, length, and position of pauses, consistency over time),^4^ will make our measures more fine-grained than they currently are—which is exactly what we need!

We need NPAs that embrace the complexity of the disorder. For example, PwMS frequently experience physical symptoms such as fatigue, visual, or motor impairments that can directly affect cognitive performance on NPA. Especially early in the disease, changes in motor functioning may be too subtle to be detected via existing coarse instruments such as the Expanded Disability Status Scale (EDSS), yet they may already impact patients’ responses significantly. Integrating fine-grained measures of visual and motor functioning (e.g. stability of trajectories in terms of directionality, velocity, or pressure) in innovative paradigms, such as virtual reality and eye-tracking, might offer possible solutions. Another promising development in this context is smartphone applications detecting changes in typing behavior, indicating disease activity.^10^ Overall, a shift toward more comprehensive assessments is crucial to effectively address the challenges posed by MS.

From a more philosophical standpoint, it may be worthwhile to re-examine the overall concept of cognition. The cultural evolution that we have been part of as society may have changed how our brains respond to stimuli from the environment. The modern world is immersed in technology, presenting new cognitive challenges such as processing information at faster speeds and differently utilizing memory and navigational skills. For instance, with the introduction of smartphones, it is no longer necessary to memorize a route and factual information can be looked up within seconds. On the contrary, we are continuously distracted by snippets of information. This may have expanded or at least changed our cognitive abilities dramatically. Therefore, to provide a more accurate assessment of cognitive functioning today, it may be necessary to develop neuropsychological tests based on new theoretical concepts that consider the demands of the modern world.

In summary, while current NPAs provide valuable information about cognitive functioning in PwMS, there is potential for improvement. By embracing digitalization, developing new and more comprehensive tests, and accounting for physical symptoms, we propose that cognitive functioning in PwMS can be assessed more efficiently and reliably. Moreover, updating norm scores and re-considering the concept of cognition appear necessary. Overall, we advocate for innovation in neuropsychological testing to benefit early and accurate identification (and possibly treatment) of cognitive impairment in PwMS.
